# Amino Acid Sensing by mTORC1: Intracellular Transporters Mark the Spot

**DOI:** 10.1016/j.cmet.2016.03.013

**Published:** 2016-04-12

**Authors:** Deborah C.I. Goberdhan, Clive Wilson, Adrian L. Harris

**Affiliations:** 1 Department of Physiology, Anatomy, and Genetics, University of Oxford, Oxford OX1 3QX, UK; 2 Weatherall Institute of Molecular Medicine, University of Oxford, Oxford OX3 9DS, UK

## Abstract

Cell metabolism and growth are matched to nutrient availability via the amino-acid-regulated mechanistic target of rapamycin complex 1 (mTORC1). Transporters have emerged as important amino acid sensors controlling mTOR recruitment and activation at the surface of multiple intracellular compartments. Classically, this has involved late endosomes and lysosomes, but now, in a recent twist, also the Golgi apparatus. Here we propose a model in which specific amino acids in assorted compartments activate different mTORC1 complexes, which may have distinct drug sensitivities and functions. We will discuss the implications of this for mTORC1 function in health and disease.

## Teaser

Cell metabolism and growth are matched to nutrient availability via amino-acid-regulated mechanistic target of rapamycin complex 1 (mTORC1). Goberdhan et al. review evidence that amino acid transporters on different subcellular compartments act as amino acid sensors in this process and discuss implications for mTORC1 function in health and disease.

## Main Text

### The Microenvironmental Sensor mTORC1 and Extracellular Amino Acids

**Figure 1:**
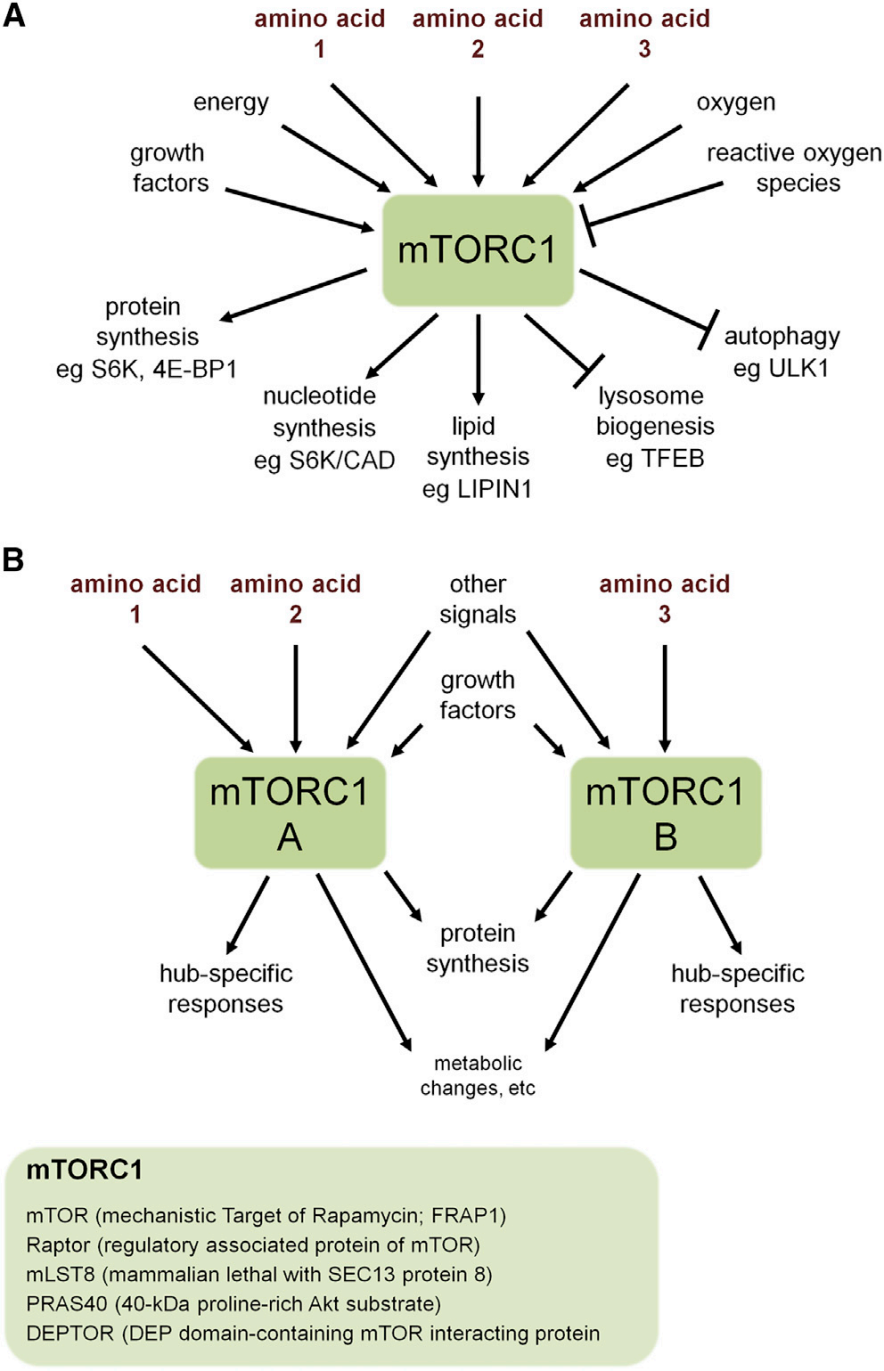
Amino-Acid-Dependent mTORC1 Signaling and Its Subcellular Control (A) Microenvironmental inputs, including specific amino acids (labeled 1, 2, and 3), are integrated by mTORC1 to control metabolic and cellular pathways that drive cell and organismal growth. CAD, carbamoyl-phosphate synthase 2 (Robitaille et al., 2013); TFEB, transcription factor EB; ULK1, Unc-51-like autophagy activating kinase 1. (B) The multi-hub model suggests that functional specificity could be achieved if distinct inputs regulate more than one mTORC1 signaling hub (labeled A and B), possibly in specific subcellular regions. Subunits of the mTORC1 complex are listed below. Note that mTOR-containing mTORC2 includes different components, such as Rictor, and regulates upstream Akt signaling (reviewed in Masui et al., 2014).

Central to the ability of a cell to adapt to its microenvironment is mechanistic (formerly mammalian) target of rapamycin (mTOR) complex 1 (mTORC1), a critical signaling hub, which is conserved from yeast to humans, regulating both growth and metabolism (Figure 1A). mTORC1 activity is regulated by a wide range of signals (reviewed in Dibble and Manning, 2013), including growth factor signaling (Gao et al., 2002, Inoki et al., 2002), cellular energy levels via AMP-dependent kinase (AMPK; Kimura et al., 2003, Inoki et al., 2002), oxygen levels (Brugarolas et al., 2004), and nutrients, particularly amino acids, as discussed below.

Genes encoding the key mTORC1 kinase component, mTOR, were first identified in yeast, as *TOR1* and *TOR2*, through molecular genetic studies, which revealed that they were important targets of the drug rapamycin (Heitman et al., 1991, Li et al., 2014). The term mTOR was initially used to refer specifically to TOR’s mammalian homologs, while nonmammalian TOR was referred to simply as TOR (Hall, 2013). However, a second definition, mechanistic TOR, has more recently also been employed in articles where mammalian, vertebrate, and invertebrate TOR are considered together, and for simplicity, we will use the latter term in this review.

Following on from the landmark studies in yeast, mTOR and its regulation by amino acids were shown to be conserved in mammals (Sabatini et al., 1994, Hara et al., 1998). A combination of genetic analysis in flies (Gao et al., 2002) and biochemical work using human cells (Inoki et al., 2002) led to a step change in thinking: growth factor signaling through PI3-kinase (PI3K) and Akt was shown to lie upstream of mTORC1 (reviewed in Goberdhan and Wilson, 2003), partly, but not exclusively, acting through the heterotrimeric tuberous sclerosis complex (TSC) and the monomeric GTPase, Ras homolog enriched in brain (Rheb).

The best-characterized downstream function of mTORC1 is the control of mRNA translation. This is achieved via phosphorylation and suppression of the translation initiation inhibitor, eukaryotic initiation factor 4E-binding protein 1 (4E-BP1), in conjunction with activation of S6 kinase (S6K), which controls the transcription of a broad range of ribosome biogenesis genes (Chauvin et al., 2014). However, mTORC1 has numerous other biochemical targets with important metabolic and cellular functions (Dibble and Manning, 2013; Figure 1A).

**Figure 2:**
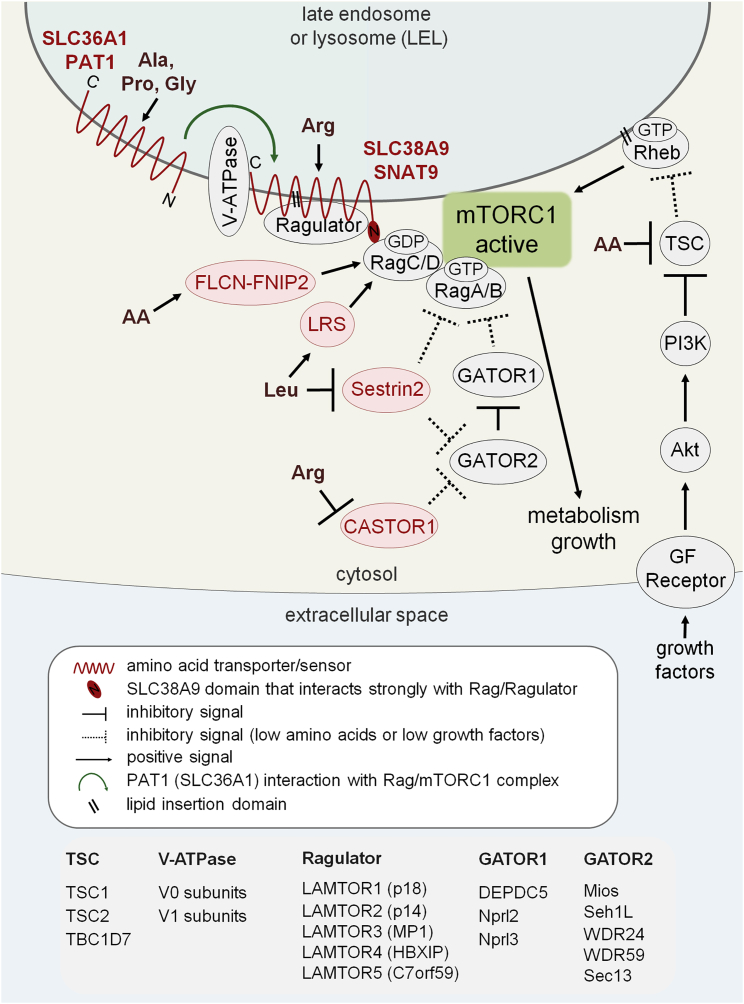
Model of Amino-Acid-Dependent mTORC1 Regulation from LELs Schematic depicts GTP/GDP loading of Rag and Rheb G proteins and protein-protein interactions taking place in the presence of amino acids and growth factors, leading to full activation of mTORC1 (green). Inhibitory interactions that happen in the absence of these positive regulators are shown by dotted crossbars. Multiple sensors are indicated in red text: the transporters (red wavy lines) SLC38A9 (SNAT9) and PAT1 (SLC36A1), and the cytosolic sensors leucyl-tRNA synthetase (LRS), folliculin and its binding partner (FLCN-FNIP), Sestrin 2, and CASTOR1 (pink ovals). They respond to specific amino acid inputs to recruit and activate mTORC1 on the surface of LELs. Green arrow indicates the interaction between PAT1 (SLC36A1) and an mTORC1 supercomplex, which is less stable than for SLC38A9. TSC localization on the LEL surface reduces amino-acid- and/or growth-factor-dependent mTORC1 signaling. Topologically equivalent extracellular space and intracellular compartment lumens are indicated in pale blue in this and subsequent figures. Subunits of mTORC1 regulatory components are listed below. Schematic adapted from model presented in Chantranupong et al. (2015).

This complex metabolic regulatory network, in the classical paradigm, is represented by a model in which microenvironmental inputs funnel through a single mTORC1 hub to give a wide range of outputs (Figures 1 and 2; Chantranupong et al., 2015). However, there is increasing awareness that many signaling cascades are controlled at a subcellular level, providing the flexibility for a more refined response within a single cell. Several studies of mTORC1 amino-acid-sensing mechanisms (Thomas et al., 2014, Jewell et al., 2015, Fan et al., 2015), discussed below, support the existence of a multi-hub model with at least two forms of mTORC1 (Figure 1B) controlled by amino acids in different parts of the cell.

What amino acids regulate mTORC1 activity? Pioneering work in Chinese hamster ovary cells (Hara et al., 1998, Beugnet et al., 2003) and in the *Xenopus* oocyte system (Christie et al., 2002) initially suggested that mTORC1 primarily responds to intracellular levels of leucine. However, subsequent studies have highlighted sensitivities to other amino acids, such as arginine, glutamine, and serine (Wang et al., 2015, Jewell et al., 2015, Fan et al., 2015, Carroll et al., 2016).

Amino acid transporters were initially implicated in mTORC1 regulation as passageways through the plasma membrane, enabling amino acids to enter cells and activate cytoplasmic amino acid sensors (Christie et al., 2002, Beugnet et al., 2003). Consistent with this, the heterodimeric amino acid transporter CD98, solute-linked carrier (SLC)3A2-SLC7A5 (CD98hc-LAT1), in combination with a glutamine transporter (SLC1A5), activates mTORC1 by exchanging leucine for glutamine to increase intracellular leucine levels (Nicklin et al., 2009). Other cell-surface amino acid transporters, e.g., LAT1 and LAT3 (Wang et al., 2013) and SLC38A2 (SNAT2; Pinilla et al., 2011), have also been linked to mTORC1 signaling. The cationic amino acid transporter Slimfast regulates mTORC1 in the adipose-like fat body of the fly larva, controlling growth of the organism in an endocrine manner (Colombani et al., 2003). These studies, however, leave unanswered the question of how amino acids are sensed once inside the cell.

### Intracellular Amino Acid Transporters as Regulators of mTORC1

**Figure 3:**
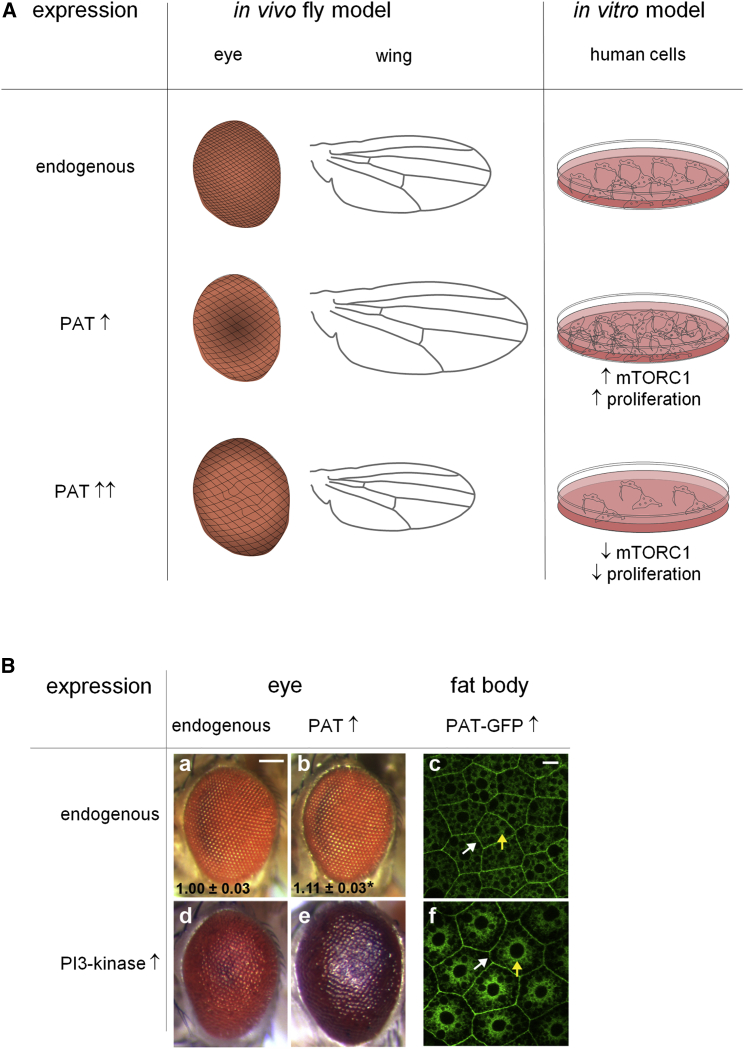
Growth Regulation by PATs (A) Overexpressing one (PAT ↑) or two (PAT ↑↑) copies of a PAT amino acid sensor in vivo has different effects on growth of developing structures in the fly. Within the postmitotic cells of the compound eye, ommatidia (unit eyes) and overall eye size progressively enlarge. In the wing, overexpression of one copy of the *PAT* gene also enhances growth, primarily through increased cell proliferation, but two copies reduce it. Similarly, using cultured HEK293 cells, modest human PAT1 overexpression in a stable cell line (PAT ↑) increases mTORC1 signaling and cell proliferation; however, signaling and proliferation decrease with transient (high level) expression, probably through a dominant-negative mechanism. (B) PAT overexpression in the fly eye leads to a mild, but measurable, increase in growth (b versus a), in contrast to loss of *PTEN*, a PI3K antagonist, which has a pronounced growth-stimulatory effect, disturbing the hexagonal array of ommatidia (d). Increased PI3K signaling significantly enhances PAT-induced growth (e versus b), promoting intracellular localization of a GFP-tagged PAT: GFP-PAT is marked at the cell surface (white arrow) and inside (yellow arrow) larval fat body cells (f versus c; images from Ögmundsdóttir et al., 2012). Scale bar, 100 μm (a, b, d, and e) and 20 μm (c and f).

Genetic screening of a broad range of amino acid transporters in flies highlighted members of the proton-assisted amino acid transporter (PAT) or SLC36 family as having a particularly potent effect in promoting growth in vivo and activating mTORC1 in a cell-autonomous manner: for example, overexpressing fly PAT family members in the developing eye or wing increases organ growth (Figure 3A; Goberdhan et al., 2005). The ability of PATs to promote growth increases significantly when growth rates and mTORC1 signaling in the eye are stimulated by activated PI3K (Figure 3B; Ögmundsdóttir et al., 2012), a signaling defect frequently associated with human cancer. Consistent with this context-dependent effect on growth, the fly PAT, Pathetic (Path), is more critical for the growth of neurons with large dendrites than those with small dendrites during normal development (Lin et al., 2015). Subsequent analysis in HEK293 and MCF-7 breast cancer cells has shown that the two broadly expressed human PATs, PAT1 and PAT4, are required for amino-acid-dependent mTORC1 activation and cell proliferation. These two human PATs can also promote growth in transgenic flies in vivo (Heublein et al., 2010).

**Figure 4:**
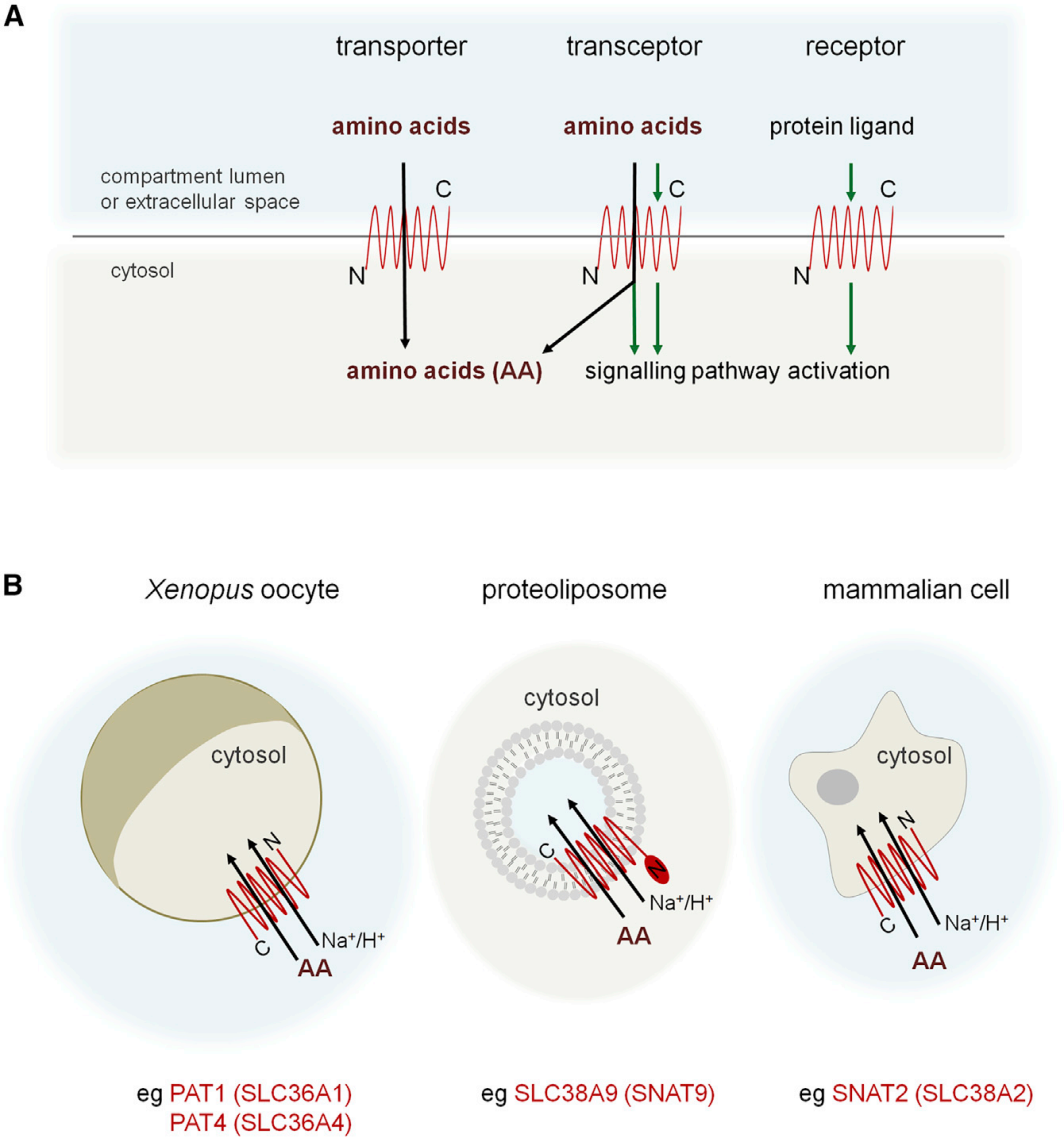
Amino Acid Transport and Sensing (A) Amino acid transporters were originally classified by their ability to translocate specific groups of amino acids across the lipid bilayer (left). However, some can activate amino-acid-dependent signaling, either in the presence or absence of transport. These so-called “transceptors” may be the precursors to modern-day receptors (right). Black arrows represent amino acid transport and green arrows signal transmission. (B) Amino acid (AA) transport has been studied in vitro using *Xenopus* oocytes, facilitated by their large size (∼1,000 μm diameter) and little background transport activity; in reconstituted proteoliposomes (up to 500 μm diameter); or in human cells (∼20 μm diameter), which may contain multiple endogenous transporters. Note that in proteoliposomes, external medium may be topologically equivalent to cytosolic side of lipid bilayer, unlike the other two models.

Before these studies, human PAT1 and PAT2 had already been shown to transport alanine, glycine, and proline by proton-coupled secondary active transport (Figure 4B; Boll et al., 2002, Chen et al., 2003). By comparison, Path expressed in *Xenopus* oocytes (Figure 4B) is not proton assisted and has a much higher amino acid affinity and lower transport capacity, at least for alanine. This and the fact that several PATs are concentrated at the surface of late endosomes and lysosomes (LELs) in many cell types led to the proposal that they might behave as intracellular amino acid sensors that activate mTORC1 through direct signaling, acting as so-called “transceptors” (Figure 4A; Goberdhan et al., 2005, Goberdhan, 2010). The cell-surface amino acid transporter SNAT2 (SLC38A2) may also be a transceptor, since it activates mTORC1 in the presence of the nonmetabolizable amino acid analog Me-AIB (Pinilla et al., 2011).

A key breakthrough in understanding the subcellular control of mTORC1 by amino acids was the finding that an activated heterodimer of Rag GTPases, RagA or RagB together with RagC or RagD, positively regulates amino-acid-dependent mTORC1 activation (Sancak et al., 2008, Sancak et al., 2010). They do this by recruitment of mTOR to the surface of compartments positive for Rab7 and LAMP2 (lysosome-associated membrane protein 2), both markers for LELs. This regulatory role of the Rags is conserved in yeast (Dubouloz et al., 2005), flies (Kim et al., 2008), and mice (Efeyan et al., 2014), where they are involved in growth control. However, despite these regulatory parallels, the yeast Rag homologs, Gtr1 and Gtr2, seem to play a different role in controlling the subcellular localization of TOR compared to their mammalian counterparts (Kira et al., 2016) and are not essential for sustained TORC1 activation (Stracka et al., 2014).

An amino-acid-regulated mTORC1-containing protein supercomplex was identified on LELs, predominantly by biochemical analyses (Sancak et al., 2010, Zoncu et al., 2011). It linked the Rags to the LEL membrane via the so-called Ragulator (LAMTOR) complex (Figure 2; reviewed by Jewell and Guan, 2013, Bar-Peled and Sabatini, 2014, Shimobayashi and Hall, 2016). Proteins regulating or within this complex have been associated with cancer. For example, components of GATOR1 (Figure 2), which negatively regulates amino acid sensing, act as tumor suppressors (Bar-Peled et al., 2013), and RagC mutations have recently been linked to follicular lymphoma (Okosun et al., 2016). Furthermore, amino acid starvation and inactivation of the Rag heterodimer have been linked to recruitment of the tumor suppressor TSC to LELs (Demetriades et al., 2014, Demetriades et al., 2016, Carroll et al., 2016), while another group has reported that TSC localization is primarily regulated by growth factor signaling (Menon et al., 2014).

But how are amino acids sensed at the LEL surface? Since the V-ATPase proton pump is part of the LEL-located mTORC1 supercomplex and its interactions are modified by amino acids, it was proposed as the sensor (Zoncu et al., 2011). The LEL-localized human PAT1, however, coimmunoprecipitates with RagC (Ögmundsdóttir et al., 2012); this led to an alternative model in which the amino acid sensing is carried out by amino acid transporters based on their ability to bind amino acids.

More recently, SLC38A9, a member of an amino acid transporter family with structural similarities to PATs, was highlighted as an amino-acid-sensitive regulator of the LEL-located mTORC1 supercomplex (Figure 2; Wang et al., 2015, Rebsamen et al., 2015, Jung et al., 2015). Coimmunoprecipitation experiments with different components of this complex consistently pull down SLC38A9, but not PAT1 or other amino acid transporters. This strong interaction is dependent on SLC38A9’s cytosolic N-terminal tail, which has a high affinity for the Rag/Ragulator complex. As discussed later, the stability of this interaction may explain why other transporters were not pulled down under the conditions employed for these studies.

SLC38A9 expressed in proteoliposomes (Figure 4B; Wang et al., 2015, Rebsamen et al., 2015) binds to several amino acids with different affinities. It potentially is involved in arginine sensing (Wang et al., 2015), though it has higher affinity for other amino acids, such as glutamine (Rebsamen et al., 2015). Like some PATs (Goberdhan et al., 2005, Pillai and Meredith, 2011) and SLC38A2 (Pinilla et al., 2011), SLC38A9 may act as a transceptor because of its close association with the mTORC1 supercomplex. The high degree of evolutionary conservation in other aspects of mTORC1 regulation, however, suggests that in the absence of a fly SLC38A9 homolog, this transporter cannot by itself resolve the amino-acid-sensing puzzle.

Where do transporters like PAT1 and SLC38A9 sense amino acids? The primary focus has been on the LEL lumen, although it is also possible that sensing at the cytosolic side could be involved. Labeled extracellular amino acids rapidly enter the LELs (Zoncu et al., 2011), probably by a combination of endocytosis and uptake across the LEL membrane. For example, LAPTM4b (lysosomal-associated transmembrane protein 4b) recruits the heterodimeric transporter SLC7A5-SLC3A2 (LAT1-CD98hc) to the lysosome, where it can promote leucine uptake (Milkereit et al., 2015). The LEL luminal microenvironment, therefore, provides a complex readout of endocytosed extracellular and transported cytosolic amino acid levels. Indeed, the versatility of LELs as amino acid sensing hubs is well illustrated by the cellular response to starvation. Reduced mTORC1 signaling induces degradation of endocytosed extracellular proteins (Palm et al., 2015, Pavlova and Thompson, 2016) and autophagy, driving breakdown of cellular proteins via autolysosome formation (Yu et al., 2010, Carroll et al., 2015): the free amino acids produced are critical in maintaining or restoring mTORC1 activity.

While amino acid transporters potentially provide a direct link between LEL luminal amino acids and mTORC1 activation, cytosolic amino acids have also been implicated in the activation process (Figure 2) and may play an important and complementary role in amino acid sensing. Leucyl-tRNA synthetase (LRS) may act as a leucine sensor that switches on RagD (Han et al., 2012). Cytosolic folliculin (FLCN) and interacting protein, FNIP, mediate activation of RagC and RagD by amino acids by acting as a GTPase-activating complex that converts the Rags into their functionally active GDP-bound forms (Tsun et al., 2013). Furthermore, Sestrin 2, a member of the Sestrin family of proteins, which activate RagA and RagB either directly or indirectly via GATOR2 (reviewed in Shimobayashi and Hall, 2016), also senses leucine (Wolfson et al., 2016). Recently, a vertebrate-specific protein, CASTOR1 (or GATS-like protein 3), has been reported as a cytosolic arginine sensor, which blocks GATOR2 function in the absence of arginine (Chantranupong et al., 2016).

Aside from these molecules that directly interact with amino acids to control mTORC1 activity, several other proteins have been implicated in the amino acid sensing process and are briefly reviewed below. Some are of particular interest because they may involve subcellular compartments other than LELs and therefore are consistent with a multi-hub model for mTORC1 control.

### Alternative Sensing Mechanisms

The MAP kinase regulator MAP4K3 was shown to be required for amino-acid-dependent mTORC1 signaling using HEK293T and HeLa cells (Findlay et al., 2007). This function is conserved in *Drosophila* and appears to involve interactions with the Rag GTPases (Bryk et al., 2010). The Class III PI3-kinase, Vps34, which catalyzes the synthesis of the lipid, phosphatidylinositol (3)-phosphate, an important regulator of endocytosis and autophagy, was identified as a potential mediator of mTORC1’s amino acid sensitivity (Byfield et al., 2005, Nobukuni et al., 2005). However, in this case, studies in flies did not suggest a role in normal growth in vivo (Juhász et al., 2008).

Two important points can be concluded from these findings. First, genetic manipulation of any molecule that interacts with and/or modifies components of the LEL-localized mTORC1 supercomplex could modulate the amino acid sensing mechanism. More elaborate experiments demonstrating direct amino acid interaction are required to identify genuine amino acid sensors. Second, when mTORC1 is regulated from the surface of intracellular compartments within the secretory and endolysosomal system, its activity will be heavily influenced by changes in membrane trafficking and endolysosomal maturation, themselves controlled by growth factor and mTORC1 signaling (Ögmundsdóttir et al., 2012, Zhang et al., 2015). Developing better cell systems to analyze this intricate regulatory interplay will be a critical objective in the coming years.

Interestingly, the identification of two other molecules involved in membrane trafficking as regulators of an alternative mTORC1 complex has recently provided evidence for a multi-hub model of mTORC1 regulation. The first, Arf1, traditionally implicated in Golgi transport, is involved in glutamine-dependent mTORC1 activation via a Rag-independent lysosomal mechanism in mouse embryonic fibroblasts and HEK293A cells (Jewell et al., (2015). The second, Rab1A, involved in ER/Golgi trafficking, affects amino-acid-dependent mTORC1 activation on the Golgi, also in a Rag-independent fashion, in HEK293E and mouse NIH 3T3 cells (Thomas et al., 2014). Rab1A is overexpressed in colorectal cancer (Thomas et al., 2014) and hepatocellular carcinoma (Xu et al., 2015), suggesting that like LEL-localized, Rag-dependent mTORC1, this alternative mTORC1 complex could be an important therapeutic target. This poses the question of how amino acids might be sensed from the Golgi apparatus, particularly since the characterized cytosolic sensors discussed above all act via the Rags.

### Amino Acid Sensing from the Golgi Apparatus

In addition to PAT1, the other widely expressed human PAT transporter required for mTORC1 activation, PAT4 (SLC36A4; Heublein et al., 2010, Matsui and Fukuda, 2013), has recently been shown to be predominantly localized on the trans-Golgi network in several cell types (Fan et al., 2015). In HCT116 colorectal cancer cells, PAT4 regulates a form of mTORC1 that appears to be resistant to the drug rapamycin and seems to more strongly affect 4E-BP1 phosphorylation than S6K, in contrast to rapamycin-sensitive mTORC1. High PAT4 levels in colorectal cancer patients are associated with poor prognosis after surgery. PAT4 interacts with Rab1A, Raptor, and mTOR on the Golgi (Fan et al., 2015), as shown by proximity ligation assay (PLA), which detects closely apposed antigens in situ in whole-mount cells (Weibrecht et al., 2010). These data support a model in which an mTORC1 signaling hub is assembled on the Golgi. The levels of PAT4 determine the resistance of mTORC1 to either glutamine or serine starvation, two amino acids that are rapidly metabolized in many cancer cells. It remains unclear whether there is any functional relationship between PAT4-regulated mTORC1 and the lysosomal form of Rag-independent, glutamine-sensitive, and Arf1-regulated mTORC1 mentioned above (Jewell et al., 2015).

Additional Rab1A biochemical and PLA data support the existence of a Rag-independent, Golgi-localized form of mTORC1 (Thomas et al., 2014). The LELs are, therefore, not the only platform for amino-acid-dependent mTORC1 signaling. The alternative mTORC1 signaling hubs potentially respond to different subsets of amino acids or different subcellular cues. For example, while LEL-located mTORC1 can detect extracellular-derived or autophagic amino acids in the LEL lumen, Golgi-localized mTORC1 could be controlled by amino acids trafficked back in a retrograde fashion from the endosomal system or brought into the Golgi lumen by Golgi-localized amino acid transporters. Significantly, the different amino acid and target preferences of specific mTORC1 hubs suggest distinct functions.

Although more studies are required to determine how different mechanisms of amino acid sensing might work together to control the mTORC1 hubs, there is a common thread in the amino acid transporter story. PAT (SLC36) and SLC38 transporter families are structurally related. The region of greatest homology is in the 11 transmembrane domain core of the transporter, which in SLC38A9 has been shown to interact specifically with components of the mTORC1 supercomplex (Figure 2; Wang et al., 2015), albeit with lower affinity than the N-terminal SLC38A9 domain. One explanation for the various, and sometimes contradictory, findings from different groups studying transporter-mediated sensing mechanisms is that the experimental approaches employed focus on different aspects of the mTORC1 regulatory puzzle. Furthermore, depending on the cell type or culture conditions employed, certain sensing mechanisms may dominate over others. There are in fact unanswered questions relating to all the mechanisms proposed to date. Consideration of how each study approaches the problem could help piece together the puzzle in a more integrated way.

### Amino Acid Sensing: The Experimental Challenges

Although for some years leucine was highlighted as the key amino acid in mTORC1 regulation (Christie et al., 2002), other important amino acids have since been implicated. In some cases, these amino acids promote cellular leucine uptake (Nicklin et al., 2009), but in others, they more directly influence mTORC1 activity. What led to the emergence of these multiple sensing mechanisms?

One factor is that different cell types, particularly cancer cells, metabolize amino acids differently, so that even nonessential amino acids, like serine or glutamine, become limiting upon starvation (Maddocks et al., 2013, Jewell et al., 2015, Fan et al., 2015). Some studies have by design, or inadvertently, tended to focus on a specific starvation regime or one mTORC1-regulatory amino acid transporter with distinct amino acid specificity, thereby probing only one sensing mechanism. Moreover, in many cancer cell lines, oncogenic pathways may selectively affect the activity of specific sensing pathways; this is illustrated by the effect of PI3K on PAT-induced growth in vivo (Figure 3B; Ögmundsdóttir et al., 2012).

Approaches for studying amino acid sensitivity also differ. Cells are often starved of amino acids and serum, and then specific amino acids are added back, or alternatively one amino acid is removed in the absence of serum (Sancak et al., 2008). Other groups have used dialyzed serum to mirror physiological conditions more closely (Averous et al., 2014, Guenther et al., 2014). These types of starvation experiments highlight requirements for particular amino acids but do not clarify whether mTORC1 is sensing different levels of specific amino acids or merely responding to amino acid presence or absence. This point can be addressed by varying sensor and amino acid concentrations (Fan et al., 2015). One important point is that although a few molecules involved in amino acid sensing have been tested in different models such as mice or flies (e.g., Kim et al., 2008, Efeyan et al., 2014, Goberdhan et al., 2005), most have not. Therefore, the physiological relevance of cell-culture starvation experiments remains uncertain when organisms employ multiple homeostatic mechanisms in vivo to maintain extracellular amino acid levels.

For those amino acid transporters that act as sensors for mTORC1, an important question has been whether they interact with the amino acid(s) that they are believed to sense. Current assays often rely on measuring amino acid transport into *Xenopus* oocytes or proteoliposomes. In the transceptor signaling model for mTORC1 regulation (Figure 4A), however, even amino acids that compete with substrates, but are not transported, are potential regulators. The known substrates of PAT1, proline and glycine, which have been implicated in cancer growth and metabolic control (Liu et al., 2015, Labuschagne et al., 2014, Li and Zhang, 2015), may therefore be sensed by this transporter (Figure 2), or other competing amino acids could be involved. In addition, neither the oocyte plasma membrane nor a proteoliposome (Figure 4B) mirrors the complex limiting membranes of LELs or Golgi, making it difficult to be certain that amino acid specificities observed are biologically significant. Amino acid affinities are typically calculated by labeling external amino acids. While the medium around an oocyte may be topologically equivalent to the LEL/Golgi lumen, the proposed site for sensing, it can represent the cytosolic side in proteoliposomes (Figure 4B; Wang et al., 2015, Rebsamen et al., 2015) and potentially provide a very different affinity profile.

The methodologies employed to demonstrate that amino acid transporters are functioning within an active mTORC1-regulatory complex influence the resulting conclusions. Coimmunoprecipitation data defined components of the mTORC1 supercomplex on LELs (e.g., Zoncu et al., 2011, Wang et al., 2015) but relied on the membrane complex involved remaining stably associated during the pull-down procedure. The PLA approach highlights less stable interactions in situ but can detect molecules separated by up to 40 nm, potentially identifying nearby proteins that are not part of a single complex. mTOR recruitment to LELs has been used as a simple assay for amino-acid-dependent activation. However, it concentrates at the surface of LELs independently of amino acids in the absence of SLC38A9 (Rebsamen et al., 2015, Jung et al., 2015). This suggests that the factors affecting recruitment are more complex than current models indicate.

Amino acid transporter overexpression has been used as a complementary approach to knockdown to demonstrate functional roles in mTORC1 activation. But the results need careful interpretation. For example, SLC38A9 overexpression makes mTORC1 signaling amino acid insensitive (Wang et al., 2015, Rebsamen et al., 2015). On the other hand, overexpression of several PATs can promote growth and mTORC1 signaling in vivo and in culture (Goberdhan et al., 2005, Heublein et al., 2010) or may, when expression levels are raised further, inhibit these processes, presumably via a dominant-negative mechanism (Figure 3A; Goberdhan et al., 2005, Zoncu et al., 2011).

These issues highlight the importance of using a range of experimental approaches when dissecting out the mechanisms of transporter-mediated amino acid sensing, particularly when considering a multi-hub model for mTORC1 control.

### New Perspective: Multiple Hubs to Integrate Different Amino Acid Inputs

**Figure 5:**
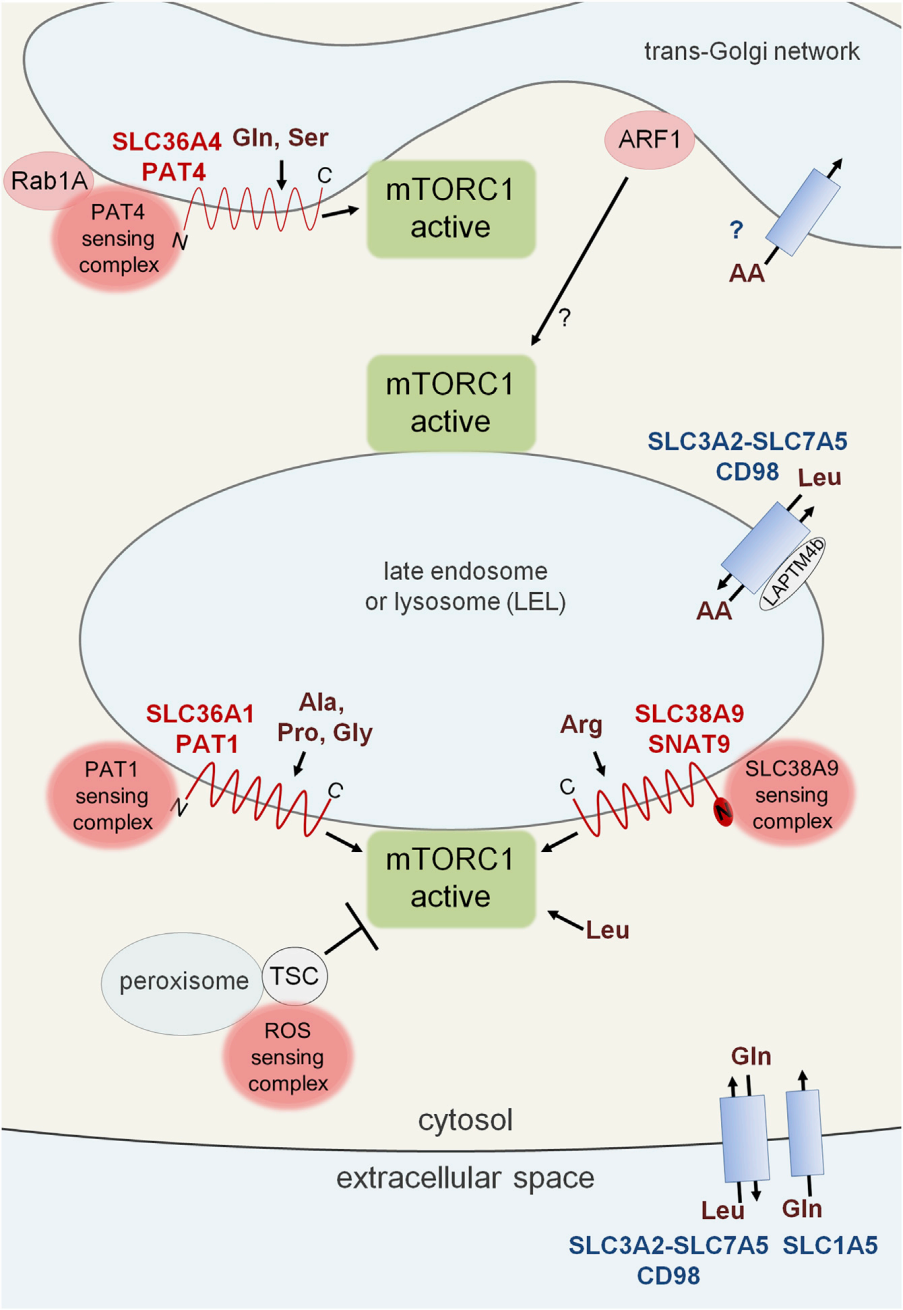
Intracellular Amino Acid Transporters and Multi-Hub mTORC1 Regulation Schematic model in which amino acid transporters (red wavy lines) act as integral components of multiple intracellular sensing supercomplexes (pink spots). These transporters sense the amino acid content of different subcellular compartments, acting in conjunction with cytosolic amino acid sensors to control the activity of specific mTORC1 hubs (green). They appear to respond to the specific amino acids indicated. Other transporters act as conduits (blue rectangles) bringing amino acids into specific compartments: for the plasma membrane, CD98 coupled with SLC1A5; and for LELs, the LAPTM4b-associated CD98 heterodimer. Equivalent conduit-like transporters for the Golgi are not yet identified. Unlike the SLC38A9- and PAT1-regulated LEL-localized sensing complexes, mTORC1 hubs controlled by ARF1 and Rab1A/PAT4 appear to be Rag independent and are therefore not under the control of the cytosolic sensors shown in Figure 2. ARF1 is typically found on Golgi membranes but is reported to control a lysosomal sensing complex. Reactive oxygen species (ROS)-activated, peroxisome-bound TSC blocks activation of mTORC1, but how this message is relayed remains unclear.

As discussed above, in vivo and cell-culture evidence strongly implicates multiple intracellular amino acid transporters in determining the amino acid sensitivity of mTORC1. Equally important, recent findings encourage thinking beyond a model where a single mTORC1 hub acts from the LELs to control metabolism and growth. Instead, mTORC1 should be considered as providing a sensory readout from more than one subcellular compartment with varying sensitivity to amino acids, potentially leading to different outputs (Figure 5).

This view complements and extends findings suggesting that additional mTORC1 signaling inputs may also be sensing metabolically important changes in other subcellular compartments (Benjamin and Hall, 2013). For example, buildup of reactive oxygen species (ROS) in peroxisomes appears to negatively regulate mTORC1 via activation of a peroxisome-associated TSC complex, ultimately promoting autophagy (Figure 5; Zhang et al., 2013). In the multi-hub model, the proposal that the amino acid microenvironment inside specific subcellular compartments could provide different readouts of a cell’s metabolic state, to which specific mTORC1 hubs respond, introduces a new layer of complexity to mTORC1 control and function. However, at present, except in the case of LELs, we have very limited understanding of how amino acids might accumulate inside different mTORC1-regulatory compartments and whether as yet unidentified compartmentalized amino acid transporters, like CD98 on lysosomes, might be involved. Moreover, even for LEL-localized mTORC1, the picture is complicated by the existence of transporter-independent sensing mechanisms for cytosolic amino acids.

The concept of different mTORC1 complexes in a multi-hub model having different target specificities, and therefore possibly distinct cellular functions, flags up the importance of testing multiple downstream targets in studying each sensing mechanism. Given the roles of mTORC1 in normal metabolism, growth, aging, and also pathological processes, such as neurodegeneration and cancer, such analysis should dissect out more specialized functions for particular amino-acid-sensing transporters and possibly reveal selective effects of different amino acids, amino acid analogs, and mTORC1 inhibitory drugs. For example, rapamycin-resistant mTORC1 preferentially affects specific mTORC1-dependent substrates and therefore potentially specific pathologies (Peterson et al., 2011, Fan et al., 2015). Ultimately, the development of better in vivo systems will be required to assess the physiological and disease relevance of the alternative mTORC1 hubs (e.g., Shen et al., 2016). The use of positron emission tomography (PET)-labeled amino acids, specific inhibitors, and/or inducible knockdown of selective regulators may be helpful in pursuing this goal. In this context, amino acid transporters now offer a selective tool to unpick the multi-hub mTORC1 model, providing new strategies for targeting of this central metabolic pathway.

## Author Contributions

D.C.I.G., C.W., and A.L.H. wrote the manuscript.
